# Genome-wide association studies of lactation yields of milk, fat, protein and somatic cell score in New Zealand dairy goats

**DOI:** 10.1186/s40104-020-00453-2

**Published:** 2020-05-25

**Authors:** Megan Scholtens, Andrew Jiang, Ashley Smith, Mathew Littlejohn, Klaus Lehnert, Russell Snell, Nicolas Lopez-Villalobos, Dorian Garrick, Hugh Blair

**Affiliations:** 1 AL Rae Centre for Genetics and Breeding, School of Agriculture, Massey University, Palmerston North, New Zealand; 2 Applied Translational Genetics Group, School of Biological Sciences, The University of Auckland, Auckland, New Zealand; 3 Research and Development, Livestock Improvement Corporation, Ruakura Road, Hamilton, New Zealand

**Keywords:** Dairy goat, GWAS, Milk production, New Zealand, QTL

## Abstract

**Background:**

Identifying associations between genetic markers and traits of economic importance will provide practical benefits for the dairy goat industry, enabling genomic prediction of the breeding value of individuals, and facilitating discovery of the underlying genes and mutations. Genome-wide association studies were implemented to detect genetic regions that are significantly associated with effects on lactation yields of milk (MY), fat (FY), protein (PY) and somatic cell score (SCS) in New Zealand dairy goats.

**Methods:**

A total of 4,840 goats were genotyped with the Caprine 50 K SNP chip (Illumina Inc., San Diego, CA). After quality filtering, 3,732 animals and 41,989 SNPs were analysed assuming an additive linear model. Four GWAS models were performed, a single-SNP additive linear model and three multi-SNP BayesC models. For the single-SNP GWAS, SNPs were fitted individually as fixed covariates, while the BayesC models fit all SNPs simultaneously as random effects. A cluster of significant SNPs were used to define a haplotype block whose alleles were fitted as covariates in a Bayesian model. The corresponding diplotypes of the haplotype block were then fit as class variables in another Bayesian model.

**Results:**

Across all four traits, a total of 43 genome-wide significant SNPs were detected from the SNP GWAS. At a genome-wide significance level, the single-SNP analysis identified a cluster of variants on chromosome 19 associated with MY, FY, PY, and another cluster on chromosome 29 associated with SCS. Significant SNPs mapped in introns of candidate genes (45%), in intergenic regions (36%), were 0–5 kb upstream or downstream of the closest gene (14%) or were synonymous substitutions (5%). The most significant genomic window was located on chromosome 19 explaining up to 9.6% of the phenotypic variation for MY, 8.1% for FY, 9.1% for PY and 1% for SCS.

**Conclusions:**

The quantitative trait loci for yield traits on chromosome 19 confirms reported findings in other dairy goat populations. There is benefit to be gained from using these results for genomic selection to improve milk production in New Zealand dairy goats.

## Background

The majority of dairy goats in New Zealand are housed and their milk is primarily used to manufacture powdered nutritional products for sale in international markets. There are estimated to be 92 farms in New Zealand milking 66,100 dairy goats. Current estimates indicate that 85% of the dairy goats belong to the Saanen breed, while Toggenburg, British Alpine, and Nubian type crosses comprise the remaining 15%. The Dairy Goat Co-operative (DGC) Ltd. is the main processor of goat milk in New Zealand, and accounts for 80% of the dairy goat production. Farms that supply DGC, and undertake herd testing, participate in an annual genetic evaluation for lactation yields of milk (MY), fat (FY) and protein (PY) and for somatic cell score (SCS). Breeding values for these traits were estimated for each animal from a multi-trait repeatability animal model using available pedigree [1].

Genome-wide association studies (GWAS) identify associations between genetic markers and phenotypic expression of traits of interest. Genetic markers are analyzed for variation across the DNA sequence of the individual’s genome [2]. A GWAS allows the statistical evaluation or association of polymorphic loci with phenotypic variance to be quantified in a given population and can provide the genetic architecture of the complex traits which can be useful in medicine, agriculture and evolution [3]. One type of genetic marker commonly used in GWAS is characterized by single-nucleotide polymorphisms (SNPs), which exhibit two or more nucleotide variants at a single base. Genome-wide association studies have been performed in many livestock species, including dairy cattle [4–6], sheep [7] and pigs [8–10]. Since release of the Illumina Caprine 50 K BeadChip (Illumina Inc., San Diego, CA), association of quantitative trait loci (QTL) in goats have been published for polledness [11], milking speed [12], wattles [13], coat colour [14, 15], supernumerary teats [16], milk production and type traits [17, 18].

Although the simplest and perhaps the most popular GWAS test for associations is between a single marker and a quantitative trait, the power of this method may suffer because a single SNP may have only low LD with the causal mutation and the LD contained jointly in flanking markers is ignored. An alternative method is to fit SNPs simultaneously using Bayesian methods, which take into account the LD between neighboring SNPs, limiting the false positive discoveries [19]. Also, the SNP sliding window approach of the multi marker methods can be used to identify the most informative genomic regions, facilitating the discovery of associated markers and possible causal mutations. In addition, SNPs can be combined into a haplotype block. Clustering SNPs into a haplotype block combines information of adjacent SNPs into composite multilocus haplotype alleles which may be more informative than individual SNPs and may also capture the regional LD information, which is arguably more robust and powerful [20–22].

Knowledge of genetic markers associated with milk production traits provides an opportunity to increase the rate of genetic gain using genomic or marker-assisted selection. Animals of above-average genetic merit can be identified at an early age and with a higher selection accuracy than conventional approaches, creating options for implementing selection schemes that reduce generation intervals [23] and increase rates of genetic gain.

To date, few GWAS have been conducted for milking traits of dairy goats. Studies that identified SNPs associated with milk production in dairy goats were performed by Martin et al. [18, 24], Palhière et al. [25] and Mucha et al. [26]. There are no published papers reporting GWAS for dairy goats in New Zealand. The objective of this study was to identify SNPs and genomic regions significantly associated with milk production traits in New Zealand dairy goats using the caprine 50 K SNP chip.

## Materials and methods

### Data

Phenotypic and pedigree records were provided by DGC from a dataset maintained by Livestock Improvement Corporation (LIC) that included estimates of 305-day lactation records for MY, FY, PY and SCS. The test interval method (TIM) (National DHIA, 2002), was used by LIC to calculate MY, FY and PY for the actual realised lactation length, or up to 305 d in milk (DIM) for those lactations with more than 305 DIM. The dataset included 106,289 animals and 236,858 lactation records. The breed composition of the goats included Alpine (592), Nubian (374), Saanen (63,370), Toggenburg (1,741) and crossbred (34,054) animals, located in the Waikato region of New Zealand. Animals were considered crossbred unless the proportion of the major breed was > 0.85. Breed composition was “unknown” for some goats (4,941). The pedigree contained 105,072 individuals spanning 5 generations, representing 1,322 sires and 27,180 dams. The records from a farm were included in the analysis if the farm supplied milk to DGC, performed herd-testing during 2017 or 2018, and contributed records for genetic evaluation. Phenotypes for the GWAS were pre-corrected for non-genetic factors using the GLM procedure of Statistical Analysis System version 9.4 (SAS) (SAS Institute Inc., Cary, NC, USA) that produced residuals after fitting the fixed effects of herd-year and parity. The significance of association between the SNP effect or haplotype effect and the phenotype adjusted for herd-year and parity, as represented by the residual, was calculated at each SNP position.

### Genotyping

Skin samples from 3,894 animals distributed in 21 herds were collected for SNP genotyping with the Illumina Caprine 50 K BeadChip (Illumina Inc., San Diego, CA). For three of the herds, only does in their first or second parity were sampled (14% of genotyped animals). Does of all parities were sampled in the remaining 18 herds (86% of genotyped animals). The recorded ancestors of the sampled animals were born between 2003 and 2015 and included 154 sires and 2,024 dams. Genotyped animals were of Saanen (1,436), crossbred (1,669), or unknown (789) breeds. A total of 51,462 SNPs were obtained.

The SNP & Variation Suite v8 (SVS) [27] software was used for quality control, principal component analysis (PCA) and one of the GWAS. Quality control was performed to remove genotypes from unreliable SNPs or animals. Records were removed for individuals with > 2% missing genotypes across all SNPs (call rate < 98% which excluded 162 animals), SNPs with > 1% missing genotypes across all individuals (call rate < 99%), that deviated significantly from Hardy-Weinberg equilibrium threshold of *P* > 10^− 6^ or had minor allele frequency < 1%. After these quality control edits, 3,732 animals and 41,989 SNPs remained for association analysis and the average distance between SNPs was 58.2 kb and the average *r*^2^ between two neighbouring SNPs was 0.15.

### Genome-wide association study

A single-SNP GWAS was performed in SNP & Variation Suite v8 (SVS) [27] software to identify SNPs significantly associated with the milk traits. The single-SNP GWAS (ssGWAS) is based on a linear regression test of the fixed covariate effect of a single marker, which treats each SNP as if it had an additive effect. Population structure was estimated by principal component analysis (PCA) in SVS using the method described by Price et al. [28]. The genomic relationship matrix was used to compute the principal components. The top 50 principal components captured 47% of the variation and were subsequently included as fixed effects in the ssGWAS method. To correct for multiple testing, a Bonferroni correction of α = 0.05 was applied to the genome-wide significance threshold (Significance threshold = α/number of SNP). The SNP effects were declared significant at a genome-wide level of *P* = 1.1 × 10^− 6^ (0.05/41,989). Quantile-quantile plots were examined to determine the validity of the *P* for the ssGWAS.

A BayesC GWAS was implemented in GenSel Software [29] fitting all SNPs simultaneously (sBayesC) to determine the proportion of variance explained by the SNPs. The algorithm uses MCMC methods to calculate samples from the posterior distributions of marker effects and variances, and inferences were made using the posterior means. The chains include 20,000 iterations after a burn-in of 1,000 iterations. For this model the priors for the genetic and residual variances were based on posterior means in a previous analysis [30]. It was assumed that 99.8% of the SNPs have no effect on the trait. The genome was partitioned into 1 Mb windows and the multi-locus contribution to genetic variance of the combined effects of SNPs within every one of these intervals were simultaneously estimated by sBayesC [19]. The 1 Mb windows that explained > 1% of genetic variance were considered to be associated with the traits.

The seven most significant SNPs clustered on chromosome 19 were combined into a haplotype block to further investigate true associations from the SNP analyses. The BayesC method was implemented a second time but with the alleles in the haplotype block included as fixed covariates while the remaining panel SNPs were fitted simultaneously as random effects (hBayesC). Thus, covariates for haplotype allele dosage were fitted instead of the dosage of alleles at each individual SNP in the QTL region. An expectation-maximization (EM) algorithm was used to estimate haplotype allele frequencies and haplotype alleles with an EM probability ≥ 50% were included in the analysis (10 of the 28 haplotype alleles).

To test for non-additive effects of the haplotype alleles, a BayesC model was re-run again in GenSel, but fitting diplotypes (pairs of haplotypes) (dBayesC). Diplotypes were defined as class effects, but were only constructed for the two most common haplotypes. The effects of these diplotypes and the remaining eight haplotypes were fitted as fixed with the remaining SNPs simultaneously fitted as random effects.

Effects of haplotypes and diplotypes on the production traits were obtained using the GLM procedure of SAS version 9.4 (SAS Institute Inc., Cary, NC, USA). The model fitted for each trait and each haplotype, was **Y**_***i***_ = **b**_**0**_ + **x**_***i***_**b** + **e**_***i***_ where **Y**_***i***_ is the residual phenotype of animal ***i***, **b**_**0**_ is the intercept, **x**_***i***_ is a row-vector indicating which haplotype and how many copies of the haplotype are carried by the animal; **b** is the effect of the haplotype and **e**_***i***_ is a residual effect. For the diplotype analysis, diplotype was treated as a class effect based on the number of copies of the two most common haplotypes.

Ensembl was used to search for genes closest to the most significant SNPs [31]. Gene annotation was retrieved if the SNP was located on an intron, lying 0–5 kb upstream or downstream from gene boundaries, or, if the SNP was located in intergenic regions, the SNPs were assigned to the closest gene.

## Results

Descriptive statistics for raw lactation yields of first and second parity genotyped does are in Table 1.

**Table 1:** Descriptive statistics of milking traits of genotyped New Zealand dairy goats in their first and second parity (*n* = 7,284)

Trait	Mean	SD^a^	Min	Max	CV^b^
Lactation length, d	272.3	117.0	60.0	696.0	43
Lactation yields					
Milk, kg	804.5	290.4	58.4	2,005.8	36
Fat, kg	26.7	10.3	2.0	76.5	39
Protein, kg	25.0	8.9	2.4	63.0	36
SCS^c^	8.6	1.3	3.5	13.7	15

^a^SD = standard deviation across herds

^b^CV = coefficient of variation

^c^SCS = somatic cell count calculated as log_2_ (somatic cell count)

**Fig. 1:**
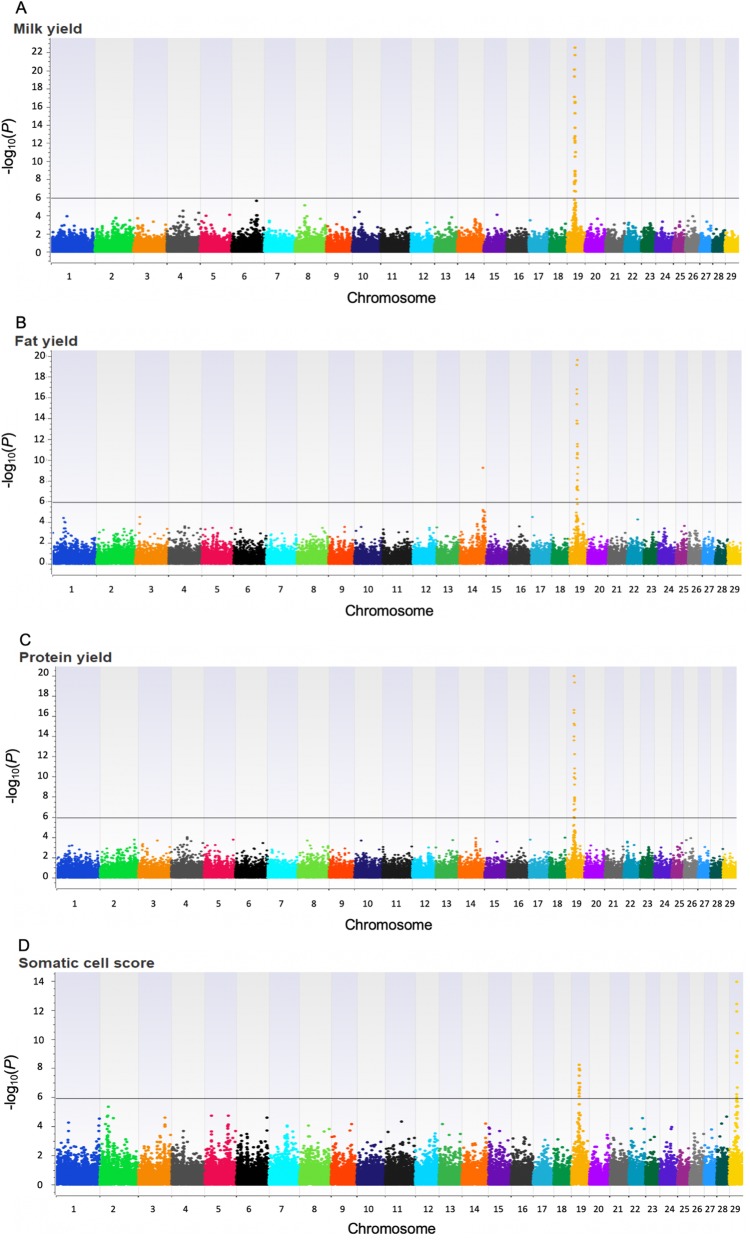
Manhattan plot of ssGWAS for lactation yields of milk (**a**), fat (**b**) and protein (**c**) and average somatic cell score (**d**), using the Illumina Caprine 50 K BeadChip (Illumina Inc., San Diego, CA) in 3,732 New Zealand dairy goats. The *P*-values [−log_10_ (*P*)] for each SNP are shown on the *Y*-axis and chromosomes 1–29 are shown on the *X*-axis. The horizontal line indicates the Bonferroni-corrected genome-wide threshold at *P*-value 0.05

Figure 1 shows the Manhattan plot for the ssGWAS for lactation yields of MY, FY and PY and average SCS. The horizontal lines represent the Bonferroni-adjusted genome-wide significance threshold. A total of 43 genome-wide significant SNPs were detected across all four traits. A highly significant region (19:24,836,694–19:28,953,102) was identified on chromosome 19 for all four traits. In this region, 26 SNPs are associated with MY, 24 SNPs associated with FY and PY and 11 SNPs associated with SCS. Another significant region was identified on chromosome 29 (29:24,850,418–29:25,328,810) with 11 SNPs associated with SCS. The two top SNPs associated with MY, FY and PY were detected on chromosome 19 (19:26,610,610 and 19:26,662,281) with significance levels of log_10_(*P*) = 22.51 and 21.67 for MY, 19.14 and 19.60 for FY, and 19.93 and 19.31 for PY. These two SNPs were also the top SNPs on chromosome 19 associated with SCS [−log_10_(*P*) = 8.22 and 7.93, respectively]. Results obtained from the ssGWAS model showed that the top SNP (19:26,610,610) explained 4.4% of the total variance for MY and 3.4% for FY and PY.

**Fig. 2:**
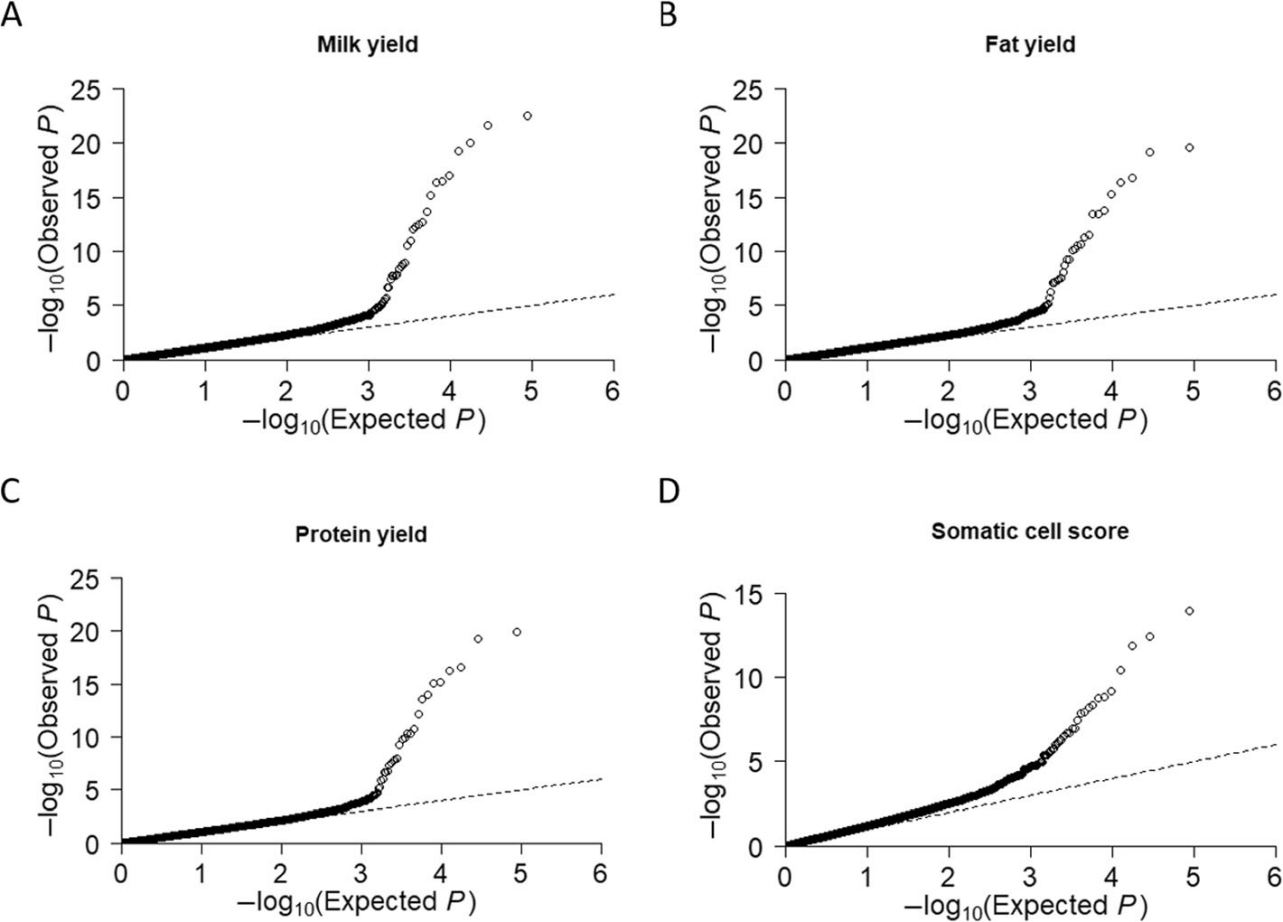
Quantile-quantile plots observed and expected *P*-values [expressed as –log_10_ (*P*)] of the ssGWAS for yields of milk (**a**), fat (**b**), protein (**c**) and somatic cell score (**d**) in New Zealand dairy goats

The Quantile-Quantile plot (QQ-plot) in Fig. 2 shows the observed and expected *P*-values [expressed as log_10_(*P*)] of the ssGWAS for lactation yields of MY, FY, PY and SCS. The dashed line represents the distribution of the SNPs under the null hypothesis that there is no association of SNPs with the trait of interest. The strong deviation of the observed from the expected *P*-values for all four QQ-plots indicate that there were more SNPs significantly associated with all of the four traits than would be expected by chance.

In this study the sBayesC model partitioned the genome into 2,520 1 Mb SNP windows with an average of 17 SNPs per window. The windows were sorted based on the proportion of genetic variance each window captured. The genomic region with the highest proportion of explained genetic variance for MY, FY and PY was on chromosome 19 (19:26,029,220–19:26,956,209). The combined effect of the 15 SNPs within this window was estimated to explain 9.62% of the genomic variance for MY, 8.09% for FY, 9.09% for PY and 0.94% for SCS. The probability that this window explained more than the average genetic variance expected under an infinitesimal model of inheritance was 1.00 for MY, FY and PY and 0.98 for SCS. Other windows of interest included one on chromosome 6 (6:86,050,148–6:86,990,478) explaining 1% of the genomic variance of MY, chromosome 14 (14:81,032,694–14:81,952,406) explaining 2% of the genomic variance of FY and a window on chromosome 29 (29:25,025,234–29:25,972,909) explaining 3% of genomic variance of SCS.

Table 2 shows the variances obtained from the Bayesian analyses in GenSel. The proportion of phenotypic variance explained by all SNPs fitted in the sBayesC model was 18% for MY, 16% for FY, 14% for PY and 20% for SCS. The genetic variances were reduced for MY, FY and PY, when the haplotypes or diplotypes in the QTL region were fitted as fixed effects (hBayesC and dBayesC models), the reduction representing the genetic variance explained by the haplotypes and diplotypes. When the haplotypes were fitted into the hBayesC model, the remaining SNPs explained 12% of the total variance for MY, 11% for FY, 9% for PY and 20% for SCS.

**Table 2:** Summary of variances estimated from BayesC GWAS for lactation yields of milk, fat and protein and average somatic cell score, using the Illumina Caprine 50 K BeadChip (Illumina Inc., San Diego, CA) in 3732 New Zealand dairy goats

Trait	Model^a^	Genetic variance	Phenotypic variance	Phenotypic variance explained by SNPs, %
Milk yield	sBayesC	12,925	73,865	18
	hBayesC	8,739	71,128	12
	dBayesC	8,484	70,893	12
Fat yield	sBayesC	13.15	83.27	16
	hBayesC	9.23	80.82	11
	dBayesC	9.06	80.53	11
Protein yield	sBayesC	8.61	63.91	14
	hBayesC	5.48	62.06	9
	dBayesC	5.38	61.89	9
Somatic cell score	sBayesC	0.28	1.45	20
	hBayesC	0.28	1.45	20
	dBayesC	0.28	1.45	20

^a^Models = sBayesC=BayesC model fitting all SNPs simultaneously, hBayesC = BayesC model fitting 10 haplotype alleles as fixed effect and remaining SNPs as random effects simultaneously, dBayesC = BayesC model fitting diplotypes of h1 and h2 as well as the 8 remaining haplotypes, and the remaining SNPs as random effects simultaneously

When the BayesC model was adjusted for the effects of the haplotypes or diplotypes (hBayesC or dBayesC, respectively), the SNPs that showed the highest model frequency were located on chromosomes 6 and 8. This suggested that all of the informative SNPs located on chromosome 19 were accurately included in the haplotype block.

The population frequency of the haplotype alleles and their diplotypes are presented in Table 3. The commonest haplotypes, h1 and h2, had estimated frequencies of 49% and 17%, respectively. Diplotypes were derived based on the occurrence of h1 and h2, of which, 79% of the population is estimated to have at least one copy of h1 and 34% of the population is estimated to have at least one copy of h2.

**Table 3:** Estimated population frequency of the 10 most frequent haplotypes, and diplotypes within the most significant region on chromosomes 19 associated with milk production in New Zealand dairy goats

	Haplotype	Frequency
Haplotype number		
h1	TCTTCTG	49%
h2	CTCCTGA	17%
h3	CTCCTTG	11%
h4	CCCCTTG	5%
h5	TCCCTTG	4%
h6	CCTCTTG	4%
h7	CCCCTGA	2%
h8	CCTCTGA	2%
h9	CCTTCGA	1%
h10	TTCCTTG	1%
Diplotype number		
h1-h0		30%
h1-h1		29%
h1-h2		20%
h2-h0		11%
h2-h2		3%
h0-h0		9%

Diplotype numbers with a h0 refers to the occurrence of any haplotype other than h1 and h2. The estimated effect of haplotypes and diplotypes on milk traits are reported in Table 4. The most frequent haplotype h1 has the greatest positive effect on MY, while h9 has the greatest effect on FY and PY. The diplotype with the greatest effect on yields includes two copies of h1 (h1-h1), of which 29% of the population is estimated to carry. The diplotype with the largest negative effect on MY and PY comprised of one copy of h2 (h2-h0), which is estimated to represent 11% of the population.

**Table 4:** Effects of haplotypes and diplotypes located within the most significant region on chromosome 19 on milk traits in New Zealand dairy goats

	Milk yield, kg	Fat yield, kg	Protein yield, kg	SCS^a^_,_ units
Haplotypes				
h1	73.6 (3.1)^b^	2.15 (0.11)^b^	1.91 (0.09)^b^	0.16 (0.02)^b^
h2	−57.0 (4.1)^b^	−1.87 (0.14)^b^	−1.54 (0.12)^b^	−0.08 (0.02)^b^
h3	−28.7 (5.6)^b^	−0.59 (0.19)^c^	−0.68 (0.16)^b^	−0.20 (0.03)^b^
h4	−59.1 (6.9)^b^	− 1.62 (0.24)^b^	− 1.64 (0.20)^b^	− 0.12 (0.04)^b^
h5	−70.0 (7.9)^b^	− 2.24 (0.27)^b^	− 1.89 (0.23)^b^	− 0.10 (0.04)^c^
h6	−5.7 (7.7)	− 0.10 (0.26)	− 0.07 (0.22)	− 0.06 (0.04)
h7	− 107.6 (20.9)^b^	−3.59 (0.72)^b^	− 2.58 (0.61)^c^	− 0.07 (0.11)
h8	−43.4 (18.9)^c^	−2.24 (0.65)^b^	−1.92 (0.55)^c^	0.01 (0.10)
h9	7.8 (13.1)	1.49 (0.45)^b^	1.04 (0.38)^c^	−0.02 (0.07)
h10	−44.7 (21.7)^c^	−2.15 (0.75)^c^	−1.05 (0.64)	0.16 (0.12)
Diplotypes				
h1-h0	53.4 (4.1)^b^	1.88 (0.14)^b^	1.61 (0.12)^b^	−0.18 (0.02)^b^
h1-h1	103.6 (3.8)^b^	3.10 (0.13)^b^	2.75 (0.11)^b^	0.05 (0.02)^c^
h1-h2	42.4 (4.6)^b^	1.17 (0.16)^b^	1.21 (0.14)^b^	−0.11 (0.02)^b^
h2-h0	−58.0 (6.9)^b^	−1.49 (0.24)^b^	−1.44 (0.20)^b^	−0.17 (0.04)^b^
h2-h2	−45.9 (13.5)^b^	−1.83 (0.47)^b^	−1.33 (0.40)^b^	−0.41 (0.07)^b^
h0-h0	−37.3 (8.4)^b^	−1.08 (0.29)^b^	−0.93 (0.25)^b^	−0.32 (0.04)^b^

^a^*SCS* somatic cell score

^b^*P* < 0.001, ^c^*P* < 0.05

Table 5 shows the 43 genome-wide significantly associated SNPs with the milk production traits and positional candidate genes (i.e. annotated genes that are nearest to each marker). Half of significant SNPs on chromosome 19 are mapped to introns, 27% to intergenic regions, 7% introducing synonymous substitutions and the remaining 17% located upstream or downstream to the closest genes. The two top SNPs from the ssGWAS, are located on chromosome 19 at 26,610,610 bp, introducing a synonymous substitution in the *RNASEK* gene (ENSCHIG00000009505) and at 26,662,281 bp, located within the intron of the *ASGR2* gene (ENSCHIG00000003690). Both SNPs were significantly associated with all four milk traits. Other SNPs included in the haplotype block were SNP 19:26,724,454, located within the intron of the *DLG4* gene (ENSCHIG00000009974), and 19:26,780,952, located downstream of the *ELP5 *gene (ENSCHIG00000010521), that were also significantly associated with MY, FY and PY. The functional annotation of SNP (19:27,854,624) resulted in a synonymous substitution in *MYH10* (ENSCHIG00000018616) and SNP (19: 28,079,607) located within an intergenic region but the closest gene being 166 kb from the *MYH10* gene. Both SNPs were significantly associated with SCS.

**Table 5:** Genes linked to the 43 genome-wide significant SNPs for yields of milk, fat, protein and somatic cell score in New Zealand dairy goats

Chr^a^	Position	Trait^b^	−log_10_(*P*)	Annotation	Gene name	Gene description
14	81,658,443	FY	9.21	Upstream	*ZNF16*	Zinc finger protein 16
19	24,836,694	SCS	6.97	Intron	*MYBBP1A*	MYB binding protein 1a
19	25,087,981	MY, PY	6.7.2–7.7	Intron	*KIAA0753*	KIAA0753 ortholog
19	25,413,768	MY, FY, PY	7.2–7.7	Intergenic	*WSCD1*	WSC domain containing 1
19	25,782,297	MY	6.7	Intergenic	*NLRP1*	NLR family pyrin domain containing 1
19	25,823,025	MY, FY, PY	9.9–12.5	Intergenic	*NLRP1*	NLR family pyrin domain containing 1
19	26,072,328	MY, FY, PY	16.3–19.3	Intergenic	*RABEP1*	Rabaptin, RAB GTPase binding effector protein 1
19	26,115,456	MY, FY, PY	6.0–7.4	Intergenic	*ZNF232*	Zinc finger protein 232
19	26,148,755	MY, FY, PY	16.6–20.1	Downstream	*ZFP3*	Zinc finger protein
19	26,192,128	MY, FY, PY	15.2–17.1	Downstream	*KIF1C*	KIF1C Kinesin family member 1C
19	26,420,506	MY, FY, PY	13.5–15.2	Intron	*ZMYND15*	Zinc finger MYND-type containing 15
19	26,542,254	MY, FY, PY	6.7.3–7.7	Downstream	None	Arachidonate 12-lipoxygenase, epidermal-type
19	26,578,775	MY, FY, PY, SCS	6.5–16.4	Intergenic	None	Arachidonate 12-lipoxygenase, epidermal-type
19	26,610,610	MY, FY, PY, SCS	8.2–22.5	Synonymous	*RNASEK*	Ribonuclease K
19	26,662,281	MY, FY, PY, SCS	7.9–21.7	Intron	*ASGR2*	Asialoglycoprotein receptor 2
19	26,724,454	MY, FY, PY	7.9–8.9	Intron	*DLG4*	Discs large MAGUK scaffold protein 4
19	26,780,952	MY, FY, PY	7.5–8.6	Downstream	*ELP5*	Elongator acetyltransferase complex subunit 5
19	27,360,768	MY, FY, PY	7.5–8.4	Intron	*CNTROB*	Centrobin, centriole duplication and spindle assembly protein
19	27,401,023	MY, FY, PY, SCS	6.3–13.6	Intron	*GUCY2D*	Guanylate cyclase 2D, retinal
19	27,480,793	MY, FY, PY, SCS	6.5–12.7	Intron	*ALOXE3*	Arachidonate lipoxygenase 3
19	27,529,983	MY, FY, PY, SCS	6.1–12.2	Intron	None	Vesicle associated membrane protein 2
19	27,558,520	MY, FY, PY	10.3–12.0	Intron	*TMEM107*	Transmembrane protein 107
19	27,605,322	MY, FY, PY	9.2–10.5	Intron	*CTC1*	CST telomere replication complex component 1
19	27,744,036	SCS	7.5	Upstream	*NDEL1*	NudE neurodevelopment protein 1 like 1
19	27,854,624	SCS	7.0	Synonymous	*MYH10*	Myosin heavy chain 10
19	28,038,645	MY, FY, PY, SCS	6.7–16.5	Intron	*PIK3R6*	Phosphoinositide-3-kinase regulatory subunit 6
19	28,079,607	SCS	7.8	Intergenic	*MYH10*	Myosin heavy chain 10
19	28,202,268	MY, FY, PY	7.1–8.9	Intergenic	*NTN1*	Netrin 1
19	28,578,424	MY	6.7	Intron	*STX8*	Syntaxin 8
19	28,730,193	MY, FY, PY	9.3–11.0	Intron	*GLP2R*	Glucagon like peptide 2 receptor
19	28,953,102	MY, FY, PY	6.8–7.8	Intron	None	Growth arrest specific 7
29	24,850,418	SCS	6.2	Intergenic	*NAV2*	Neuron navigator 2
29	25,175,690	SCS	11.9	Intron	*ZDHHC13*	Zinc finger DHHC-type containing 13
29	25,206,548	SCS	12.4	Intron	*ZDHHC13*	Zinc finger DHHC-type containing 13
29	25,328,810	SCS	14	Intergenic	*PTPN5*	Protein tyrosine phosphatase, non-receptor type 5
29	25,366,901	SCS	8.3	Intergenic	*PTPN5*	Protein tyrosine phosphatase, non-receptor type 5
29	25,649,038	SCS	8.8	Intron	*LDHC*	L-lactate dehydrogenase C chain
29	26,381,310	SCS	6.0	Intergenic	*OR10D3*	Putative olfactory receptor 10D3
29	26,502,551	SCS	8.8	Intergenic	None	Olfactory receptor 145-like
29	27,144,973	SCS	10.4	Intron	*OR8B4*	Olfactory receptor family 8 subfamily B member 4
29	27,407,592	SCS	6.7	Intergenic	*PANX3*	Pannexin 3
29	27,967,983	SCS	9.2	Intergenic	*PKNOX2*	PBX/knotted 1 homeobox 2

^a^Chromosome, ^b^*MY* milk yield, *FY* fat yield, *PY* protein yield, *SCS* somatic cell score

Of the 11 SNPs on chromosome 29 significantly associated with SCS, 36% were mapped to introns and 64% were in intergenic regions. The most significant SNP on chromosome 29 (29: 25,328,810) is located in an intergenic region and is 60 kb from the closest gene, *PTPN5* (ENSCHIG00000008345). Another significant SNP (29: 25,366,901), is also near the same gene (22 kb). In addition, two SNPs (29: 25,649,038 and 29: 27,144,973) significantly associated with SCS were located within introns of the *LDHC* gene (ENSCHIG00000013476) and *OR8B4* (ENSCHIG00000012776), respectively. The two remaining SNPs on chromosome 29 (26:25,175,690 and 29:25,206,548), were located within introns are of the *ZDHH13* gene (ENSCHIG00000024992).

## Discussion

Genome wide association studies have been used to identify associations between genetic markers and candidate genes for traits of economic importance. This study evaluated the associations of 41,989 SNPs with MY, FY, PY and SCS from 3,732 New Zealand dairy goats.

The ssGWAS identified 43 SNPs significantly associated with MY, FY, PY and SCS in this population. A cluster of highly significant SNPs were identified on chromosome 19 for all four traits and on chromosome 29 for SCS. The two strongest signals were identified at SNP 19:26,610,610 and 19:26,662,281. These two SNPs were in high LD (*r*^2^ = 0.94) and it is highly probable that these SNPs were in high LD with a QTL or causal variant that had a very significant effect on MY, FY and PY in this dairy goat population.

Quantile-quantile plots (Fig. 2) of the observed and expected *P*-values of the ssGWAS for each trait indicated that a large proportion of the observed *P*-values were clearly more significant than expected under the null hypothesis. This suggested there were some true associations between SNPs and genes controlling these traits.

The main advantage of the ssGWAS is the ease of significance testing. However, single-SNP analysis relies on LD between the marker and QTL, therefore the results do not provide information about the location of the causal variant, instead they correspond to the location of the marker. Also, fitting SNPs individually may result in the same signal picked up in multiple single SNP tests, thus overestimating the number of actual QTLs detected. And finally, although a significant signal is identified, if a trait is controlled by many QTLs, which is the case for most quantitative traits, the single-locus tests may prove inaccurate compared with methods where grouped (haplotypes) or all SNPs are jointly considered. For these reasons, an additional analysis was performed fitting all SNPs simultaneously in a BayesC GWAS.

The BayesC GWAS that fits all SNPs simultaneously, can improve the accuracy of detecting QTLs [32] and the 1 Mb window variances provide greater insight for identifying the genomic region of the casual variant [19] and estimates the proportion of variance explained by the SNPs.

In the Bayesian analysis, the percentage of genetic variance explained by 1 Mb genomic windows are used to make inference about the proportion of variance explained by a QTL and whether the QTL bleeds over multiple windows. The genomic window that explained the greatest level of genetic variance (8–9%) for MY, FY and PY included 15 SNPs and ranged from 26,420,506 to 26,780,952 bp on chromosome 19. Two of the SNPs located in this window were also the most frequent SNPs included in the model (suggesting they are informative SNPs that contribute to the model) and were the top SNPs identified in the ssGWAS to be associated with MY, FY and PY.

Combing these results provides strong evidence that those SNPs with the highest model frequency within the genomic window on chromosome 19 with the largest effect, are likely to be in LD with the causal variant.

To learn more about this potential QTL on chromosome 19 the seven most significant SNPs identified in the ssGWAS were combined into a haplotype block and the Bayesian analysis was re-run but adjusting for the SNPs in the haplotype block. Fitting covariates for haplotype alleles rather than the SNP alleles provides higher LD between causal mutations and haplotype alleles as the multilocus haplotype takes into account not only the LD information from the SNPs within the haplotype but also other important polymorphisms within the QTL cluster region. In addition, the use of haplotypes can provide information regarding the genetic determinants that cannot be captured by the biallelic markers. For example, when a SNP is fit in the model there is no guarantee that its alleles are in high LD with the QTL allele, whereas in the haplotype, provided there are enough SNPs to represent them, at least one haplotype must contain the favourable QTL allele and at least one must include the unfavourable allele. When the haplotypes were fitted into the hBayesC model as a fixed effect, there were no other signals on chromosome 19 of large effect, indicating that the majority of the QTL was indeed captured within the genomic region of that haplotype block. Also, the genetic variance from the hBayesC model was lower than the sBayesC, indicating that the seven SNPs located in the haplotype are capturing the variation that exists in that genomic region.

The haplotype effects on milk production in this dairy goat population were estimated for the 10 haplotypes. Haplotypes h1 and h9 had the greatest positive effect on the milk traits. Animals that carry one copy of h1 or h9 are estimated to produce + 73.6 and + 7.8 kg milk, + 2.2 and + 1.5 kg fat and + 1.9 and + 1.0 more protein, respectively, per lactation, compared with the average of the population. Both h1 and h9 are the only haplotypes that contain the T allele at the loci 19:26,610,610, which had the strongest signal on the milk traits as well as the C allele at the loci 19:2,666,281, which had the second strongest signal on the milk traits. This suggests that an animal carrying the T and C alleles at the corresponding loci will have the greatest yields per lactation compared to the average of the population. The positive effect of these loci on milk production traits should be used in combination with performance and pedigree information to generate more accurate breeding values. When selecting animals for breeding replacements, genotyped males carrying the desirable alleles can be used for mating to females to produce replacements that carry the desirable alleles and thus the potential to be high yielding animals.

When haplotypes are fitted in the model as dosage covariates we assume the haplotypes have an additive effect, which may not be true. Therefore, to test whether the effect of the haplotype block was truly additive, we fit diplotypes (pairs of haplotypes) into the model. Fitting diplotypes allows the estimation of the effect of the heterozygote without assuming it is intermediate between the opposite homozygotes, which can determine whether that haplotype allele is additive, or dominant or over-dominant etc. Results from the diplotype analysis showed that animals with the h1h2 haplotype indeed had a greater effect than the h2h2 diplotype animals, demonstrating that the h1 haplotype does have an additive effect on milk production traits in New Zealand dairy goats.

The diplotypes included in the trend regression were derived from the two most frequent haplotypes in the population, h1 and h2. The predominant diplotype (29%) in the study population had two copies of h1. Animals that do not carry either h1 or h2 had an average effect of − 37.3 kg milk per lactation, relative to the population average. If an animal has only one copy of h2 then they will have − 58.0 kg, which is 20.7 kg less than animals with neither h1 nor h2. If an animal carries one copy of h1, they will have + 53.4 kg milk, producing 90.7 kg more than an animal that carries neither h1 nor h2. If an animal carries two copies of h1 then it will have + 103.6 kg milk than the population, producing an extra 50.2 kg milk more than an animal with one copy of h1. These results follow a similar trend for FY and PY and suggest that h1 has a positive effect on milk traits and can lead to increased productive value of dairy goats in New Zealand.

Several studies have identified QTL significantly associated with milk production traits in goats (Table 6). Results from our study confirmed the presence of a QTL reported on chromosome 19 for MY, FY, PY and SCS and on chromosome 29 for SCS. In addition, several novel regions were identified, including a QTL for FY on chromosome 14 and genetic regions associated with MY, FY and PY on chromosome 23 and SCS on chromosome 5.

**Table 6:** Reported QTL associated with milk production traits in dairy goats

Trait	Chromosome	Reference
Milk yield	6, 8, 14, 19 and 21	Roldán et al. [33], Maroteau et al. [17], Martin et al. [18], Mucha et al. [26]
Fat yield	2, 14 and 19	Maroteau et al. [17], Martin et al. [18]
Protein yield	19 and 20	Maroteau et al. [17], Martin et al. [18]
Fat content	6, 7, 14, 20, 21 and 25	Roldán et al. [33], Maroteau et al. [17], Martin et al. [18]
Protein content	1, 3, 5, 6, 11, 20, 21, 28	Roldán et al. [33], Maroteau et al. [17], Martin et al. [18]
Fatty acid	1, 7, 8, 11, 14 and 29	Maroteau et al. [17]
SCS	19, 29	Maroteau et al. [17], Martin et al. [24]
Morphology traits	29	Maroteau et al. [17], Martin et al. [24]

The QTL on chromosome 19 that was strongly associated with all four traits, was reported in the French Saanen dairy goat population [12] and a mixed breed population [26]. In addition to milk traits in dairy goats, this highly significant region was also associated with type traits [24], udder floor position [12], functional longevity [25] and semen production [34], suggesting a pleiotropic QTL effect. Further investigation into this genomic region (chromosome 19, 25–29 Mb) revealed that the SNPs significantly associated with MY in the current study were different to the SNPs identified by Mucha et al. [26] in their mixed breed goat population. This could be because both studies analysed mixed breed populations, thereby having different levels of linkage disequilibrium [35], thus, the loci on the SNP have different levels of linkage disequilibrium with the unknown causal. With that said, although the individual SNPs differed in statistical significance between the goat populations, this highly significant region identified in both studies suggests the segregation of a common gene that has a major effect on milk production in dairy goats.

In this study, the most significantly associated SNP (19:26,610,610) was located on chromosome 19 introducing a synonymous substitution in the *RNASEK* gene (ENSCHIG00000009505). RNASEK is a transmembrane protein ubiquitously expressed and highly conserved across mammals. RNASEK localizes to the cell surface and endosomal pathway and closely associates with the vacuolar ATPase (V-ATPase) proton pump. RNASEK is required for endocytosis that prevents the replication of multiple pathogenic viruses such as rhinovirus, influenza A and dengue [36]. This most significant SNP was strongly associated with all four milk traits, but no previous studies have reported this SNP or any association with this gene in goats. However, this SNP is in strong LD (*r*^2^ = 0.94) with SNP 19:26,662,281, which was also strongly associated with all four milk traits. This SNP (19:26,662,281), is located within the intron of the *ASGR2* gene (ENSCHIG00000003690) and is in the same region where Mucha et al. [26] reported a loci (19:26,150,581) that is strongly association with udder depth of mixed breed dairy goats. The *ASGR2* gene encodes a subunit of the asialoglycoprotein receptor involved with the glycoprotein metabolic process, lipid homeostasis and the regulation of protein stability. Therefore, the possibility of the *ASGR2* genes involvement with the milk production traits is supported by its activity in lipid homeostasis and protein stability.

Another signal strongly associated with all four milk traits is SNP (19:27,480,793) which is located within the intron of the *ALOXE3* gene. A SNP (19:26,972,244) in the same gene region was reported by Mucha et al. [26] to be associated with udder depth of mixed breed dairy goats. This gene is part of the lipoxygenase family of enzymes and is involved in the metabolic pathway during formation of the epidermal barrier [37]. As this process includes the activity in cell differentiation, cell proliferation and fat metabolism, it is possible that this gene is involved with udder conformation [26], and subsequently milk production.

Another association which was reported by Mucha et al. [26] was for SNP (19:26,066,457), which is located near the *ALOX12* gene (GOAT_ENSP00000251535) and has a significant effect on MY in dairy goats [26]. However, this SNP and chromosome region were not significantly associated with milk production traits in this current dairy goat population.

In the current study, the SNP (19:26,192,128) was significantly associated with MY, FY and PY and is located downstream from the *KIF1C* gene (ENSCHIG00000000772). This gene is involved in the movement of molecules from the Golgi back to the endoplasmic reticulum. This SNP was also reported at the genome-wide significance level, to be associated with functional longevity in Saanen dairy goats [25]. In the same population, Martin et al. [18] also reported the same genomic region to be associated with milk production. This is not surprising as multiple studies have published a positive genetic correlation between milk production and longevity in dairy goats [38, 39].

Two significant SNPs were mapped within and close to the *MYH10* gene (ENSCHIG00000018616) which is involved in mitotic cytokinesis. The SNP (19: 28,079,607) causes a synonymous substitution and the 19: 28,079,607 SNP is located 166 kb from the gene. Both SNPs were significantly associated with SCS and not with the other milk traits.

Other genes on chromosome 19 associated with milk production traits in dairy goats include the *GH1* gene located in the 47 Mb region [40, 41] and the *STAT5A* gene located in the 42 Mb region [42]. However, in our study there were no associations for milk traits detected in these regions.

We identified a peak of significant SNPs on chromosome 29 associated with SCS. It is evident there is a QTL located on this chromosome for SCS as we detected strong signals for 11 SNPs from the ssGWAS. However, further investigation using Bayesian methods would provide more information about the genomic region of the QTL and the level of variance it explains. Previous studies have reported a chromosome-wide significant SNP on chromosome 29 associated with MY [26] and fatty acid composition [17] in French dairy goats and associated with gastrointestinal nematode resistance in dairy goats in Zimbabwe [43].

Two of the top SNPs associated with SCS (29: 25,175,690 and 29:25,206,548) are within introns of the *ZDHHC13* gene (ENSCHIG00000024992), which is associated with signal transducer activity and palmitoyltransferase activity. Palmitoyltransferase is important for the positive regulation of I-kappaB kinase/NF-kappaB signalling, which is an inflammatory signalling pathway. This gives credibility to the SNP being associated with SCS in this study.

Other genomic regions that may be involved in SCS include the *LDHC* gene, which is involved in carbohydrate metabolic processes such as the chemical reactions and pathways resulting in the formation of ATP, a universally important coenzyme and enzyme regulator. And the *OR8B4* gene, which changes the activity or state of a cell in response to a chemical stimulus by chemoreceptors i.e. smell perception.

Only one genome-wide significant signal was detected on chromosome 14 (14:81658443) with FY. Although associated with FY, the genome-wide significant SNP was not located in the immediate region of the *DGAT1* gene, a gene known to have a major effect on milk fat content in goats [18] and cattle [44]. Instead, the SNP was located upstream of the *ZNF16* gene (ENSCHIG00000020215). Although not studied in goats, this gene promotes cell proliferation and inhibits cell apoptosis in humans [45].

Despite only a few papers reporting GWAS studies in dairy goats, candidate genes related to milk traits have been widely studied. Some polymorphisms associated with milk production in goats include the *LALBA* gene (chromosome 5) which is linked to milk yield, lactose content and milk coagulation properties [46], the *MTHFR* gene (chromosome 6) involved in milk protein synthesis [47], the β-lactoglobulin gene (chromosome 11) [48, 49] associated with milk yield and daily fat and protein yield, the *TLR2* gene (chromosome 17) which is important in the recognition of the innate immune system of mastitis causing bacteria [50] and the *PRLR* gene (chromosome 20) [51] and the *STAT5A* gene (chromosome 19) [42], both which are associated with milk yield. But none of the significant SNPs in the current study were located in the regions of these genes.

Although numerous studies have provided evidence of polymorphisms within specific genes influencing milk production, there are limited studies using GWAS methodologies to identify QTL for milk production traits in dairy goats. All of the previous GWA studies identified SNPs that were of varying significance levels for different breeds [18, 24–26]. In our study, the goats were of mixed breeds, representative of the New Zealand dairy goat population.

Results from the GWAS strongly show a QTL located on chromosome 19 and the trend regression analysis suggest this is biallelic with h1 containing the desirable allele. This was detected when analysing the effects of the haplotypes and confirmed by the estimated diplotype effects. All diplotypes containing h1 resulted in positive effects on milk traits, while every diplotype that contained h2 had negative effects on the milk traits, compared with the average of the population. The fact that animals carrying one copy of both h1 and h2 still had positive effects on the milk traits shows the greater magnitude of the positive effect of h1 over the negative effect of h2.

The results from this study provide evidence that there is a likely QTL strongly associated with milk traits in this population. It is possible that the QTL has an additive effect and is biallelic. In addition, it is concluded that this QTL has a pleiotropic effect as it has been identified in other goat populations and associated with a range of traits besides milk production traits.

Although the study population was small, the significant regions identified were also reported in other studies, which gives confidence in the results. Nevertheless, the results require validation. If the new results are consistent with the current results, the identified markers could be used for marker-assisted-selection. This will enable the prediction of genetic and phenotypic value of individuals. For example, to predict the future phenotypes of offspring so that those with the best breeding values can be selected as parents of the next generation [3]. At the same time, the information on the genomic regions found in this study, can be used to facilitate the identification of candidate genes for these milk traits. Doing so would enable a greater understanding of the biology underlying the response from genomic selection, and managing possible consequences of selecting for mutations with undesirable pleiotropic effects [52]. Ultimately, these results provide an opportunity for adopting genomic selection within the New Zealand dairy goat population. Implementing genomic selection will increase the accuracy of predicted genetic and phenotypic values and reduce the generation interval, leading to increased rates of genetic improvement within this dairy goat population.

## Conclusion

The study identified one region strongly associated with milk production traits in New Zealand dairy goats. The highly significant region identified on chromosome 19 was also reported in French dairy goat populations and suggests a major pleiotropic QTL for milk production traits in dairy goats. The significant SNPs will increase the accuracy of predicted genetic and phenotypic values of individuals to allow for genomic selection. The results demonstrated in this study require validation using a larger dataset before implementing genomic selection within the New Zealand dairy goat population.

## Data Availability

Authors do not wish to share the data due to the propriety nature of the data.

## Abbreviations

dBayesC: hBayesC model but also including diplotypes
DGC: Dairy Goat Co-operative
DIM: Days in milk
EM: Expectation-maximisation
FY: Fat yield
GWAS: Genome-wide association study
hBayesC: sBayesC model but also including haplotypes
LD: Linkage disequilibrium
LIC: Livestock Improvement Corporation
MY: Milk yield
PCA: Principal component analysis
PY: Protein yield
SAS: Statistical Analysis System
sBayesC: BayesC model fitting all SNPs simultaneously as random effects
SCS: Somatic cell score
SNP: Single-nucleotide polymorphism
ssGWAS: Single-SNP GWAS
SVS: SNP & Variation Suite
TIM: Test interval method
QTL: Quantitative trait loci
